# Death of Dr. Chapman

**Published:** 1853-07

**Authors:** 


### DEATH OF DR. CHAPMAN.

The great men of our generation—the physicians, statesmen, and warriors who have shaped the age in which we live, are rapidly passing away. Wellington, Clay, Calhoun, Webster, Drake, Orfila, Pereira, Graves, are names that now belong to history. It is but a little while since we announced the death of Dr. Horner, for a long time one of the noted medical men of our country; and now we have to record the death of Dr. Chapman, who, for many years, stood confessedly at the head of the American medical profession. The death of this distinguished individual took place in Philadelphia, on the 2nd of this month.

Dr. Chapman enjoyed a reputation such as few men have the good fortune ever to gain. He was eminent as a writer, practitioner and teacher. Called early in life to a chair in the most renowned medical school of his country, he soon became one of its chief ornaments. Without the learning or the industry of many of his cotemporaries, by the charm of his eloquence and the fascination of his manners, he won his way to the very front rank of his profession. Chapman is a name associated with wit, humor, conviviality—with eloquence, kindly social impulses, and noble generosity.

Dr. Chapman, with two or three exceptions, had been longer before the public than any medical man in our country who survives him. Dr. Coxe and Dr. Caldwell are the only two, we believe, who entered public life in advance of him and are still living. He attained to a good old age, and was fortunate, not only in the length, but in the circumstances of his life. As a physician he was much confided in; patients visited him from every part of the Union. As a teacher he was admired, applauded and revered; and the evening of his life was solaced by the kindness of "troops of friends." For several years before his decease he had lived in retirement, having resigned his chair in the University when he began o feel the infirmities of age. We saw him in Baltimore at the second meeting of the American Medical Association, of which he was then President, and listened with pleasure to his address on the occasion. No one could fail to be struck with his exceedingly imposing manner. He was then in excellent health, and evinced as keen a relish for society, and as lively a concern for the honor of his profession as ever he had done.

The intelligence of Dr. Chapman's death reached us just as the Journal was going to press, and we have not leisure or space for a more extended obituary notice in our present number. A sketch of his life and services will be given at some future day.

